# Missouri Valley Veterinary Association

**Published:** 1900-04

**Authors:** J. B. Wright

**Affiliations:** Secretary-Treasurer


Secretary.

### MISSOURI VALLEY VETERINARY ASSOCIATION.

The twenty-third regular meeting was held in the Kansas City Veterinary College, Kansas City, Mo., on the evening of February 19th, at 7.30 o'clock. It was arranged to have a surgical clinic on the afternoon of the same day, but owing to the absence of subjects no clinic was held. Owing to the absence of the President, Dr. Forbes, the meeting was called to order by the Second Vice-President, Dr. Schaeffer, with the following officers, members, and visitors present: Drs. Schaeffer, Wright, Milnes, Washburn, Bennet, Kaupp, Cooper, Buckley, Saunders, Moore, and Stewart, with Drs. Falsetter and Knight (from Texas), George, Boise, and Lawton; also Messrs. Lomax, Painter, Mahon, Lawrence, Starr, Kampochmidt,

McFarland, Parker, Carswell, Burgett, Philips, Moberly, Roach, Reber, Rice, Makins, Miller, Barnhart, Immerman, Crickett, Mooney, Biggs, and Halstead. The minutes of the previous meeting were read and approved.

The Censors having reported favorably upon the application for membership of Albert Long, it was moved by Dr. Stewart that the rules be suspended and the Secretary instructed to cast the ballot for the Association; motion carried. The Secretary cast the ballot for the Association, and the candidate was duly elected. The resignation of Dr. Frank C. McCurdy was read, and on motion was accepted.

No other business being on hand, the literary programme was then begun, which was as follows:

"In-breeding," by Dr. L. Bennet; discussion led by Dr. Ross Cooper. "Clippings from German Veterinary Journals," by Dr. Washburn; discussion led by Dr. Stewart. "Scientific Horseshoeing," with exhibition of specimen shoes, by Dr. Charles Saunders. All the papers brought forth a great deal of good and interesting discussion, and especially with the paper of Dr. Saunders; it was eagerly listened to, and a great many new and valuable points were obtained. The specimen shoes Dr. Saunders had on exhibition were a rare treat to all who saw them.

After the literary programme a vote of thanks was tendered the essayists of the evening for all their excellent papers. By vote the Society decided to meet in St. Joseph, Mo., in June.

J. B. Wright,
Secretary-Treasurer.

